# Cerebro-Spinal Meningitis, or Epidemic Pneumonia

**Published:** 1870-08

**Authors:** D. A. Sheffield

**Affiliations:** Apple River, Illinois


ARTICLE XX.

### CEREBRO-SPINAL MENINGITIS, OR EPIDEMIC PNEUMONIA.

By D. A. SHEFFIELD, M.D., Apple River, Illinois.

During the last Spring, a deep impression has been made upon my mind, which has terminated in a question, an answer to which I am unable to find, either in books treating upon the subject, or the judgment of my fellow-laborers. The question is this: Is it possible that Congestion of the Lungs, with complications, may become Epidemic? It was my intention to have brought this question before the State Medical Society at its last session, but a desire to listen to the opinions of older and more experienced men, on topics of vital interest to the profession, determined me to submit the matter to you privately. The causes which have united to raise this inquiry in my mind, were the loss of quite a number of patients—all children—within a few weeks of time, from what I cannot avoid believing to have been Epidemic Pneumonia, complicated by Cerebro-Spinal Meningitis. All the patients I was called upon to visit during a period of about three weeks, and who had been first attacked by Pneumonia, invariably showed symptoms so strongly resembling those of which I had read of as presenting in Cerebro-Spinal Menengitis (never having seen an unmixed case), that, although I could not obtain a *post mortem* examination, I have no doubt of the pathological condition. A larger ratio of mortality attended these cases than I have ever had in any epidemic before. The history of one case will be sufficient for the whole, as their similarity was very remarkable.

Ella J——, aged 7 years, seized on the night of February 8th, 1870, with what was thought to be ordinary Congestion of the Lungs. Was called to see the case almost immediately after the attack. Symptoms: Extreme dyspnœa; pulse 120; tongue moist, and covered with a thin cream-colored coating; skin moderately dry; urine scanty and high colored; a suppressed, muffled cough, so constant as to keep the patient in constant agitation. Gave as follows:

℞. Sub. Mur. Hyd. .................... 6 grs.

Pulv. Ipecac et Opii, } āā, ..........12 grs.

Potassa Nit. Pulv., }

Fiat in Chart., .................... 6 grs. M.

Dose—One powder every fourth hour, alternating with *three* drops of tinct. veratrim viride in one-half a teaspoonful of spts. nit. dulcis. Also ordered a corn-meal poultice, into which had been stirred pulv. pimento, to be placed over the whole anterior part of the chest. This course was pursued for twenty-four hours, at the end of which time, saw the patient again. Symptoms: Tongue heavily loaded with a coating of a brownish color; pulse 130; urine passed twice, but scanty; lips parched and cracking; eyes suffused and secretions rapidly accumulate in the inner canthi; sub-mucous ronchus over almost the whole extent of both lungs, yet the cough is dry and harassing.

Treatment: Ordered, immediately, a full dose of castor oil, with one-half a drachm of oil of turpentine. Also gave the following:

℞. Sub. Mur. Hyd., .................. 6 grs.

Pulv. Ipecac et Opii, } āā, .........12 grs.

Potassa Nit. Pulv., }

Antimony et Pot. Tart., ............ss. grs.

Fiat in Chart., .................... 6 grs. M.

Dose—One powder every fourth hour; continue tr. veratrum as before, except that the dose be increased to *four* drops. The corn-meal poultice, containing pulv. pimento as an irritant, continued over the anterior part of the chest.

The following day, gave about the same train of symptoms, excepting that *sordes* collected rapidly on the lips and teeth. Patient perfectly rational when awake, but muttering while asleep.

*February 11.*—Patient suddenly began to exhibit symptoms of cerebral disturbance. The pupils do not readily respond to light; head continually rolling from side to side; the hands in constant motion; severe pain in the neck and occipital region, complained of only at times; tongue and secretions much the same as on the two previous days. Treatment as follows:

℞. Tr. Rhei,------------------------------ ℨj.

Magnesia Carb.,------------------------ ℨj.

Syr. Ipecac,--------------------------- ℥ss.

Spts. Nit. Dulcis---------------------- ℨjss.

Mix. Dose—One teaspoonful every three hours. The whole length of the spine, from occiput to sacrum, ordered to be rubbed freely and frequently with the following:

℞. Oleum Olivia,------------------------ ℥jss.

Oleum Succini,------------------------ ℥ss.

Oleum Caryophylli,-------------------- ℥j.

Aqua Ammonia, } āā,---------------- ℥ss.

Spts. Camphora, }

Mix.

*February 12.*—Symptoms indicate no improvement in the condition of the patient; cerebral disturbance greatly increased; pupil immovable to strong sunlight; patient continually in a comatose state; natural evacuations all ceased. Treatment: Ice-bags to be applied to the head; sinapisms to the feet; and a cantharides blister to the neck, reaching from the occiput to the first dorsal vertebra. From this time the patient gradually sank into profound coma, in defiance of all the medication my counsel and myself could devise, until death occurred on the seventh day after the attack. On the day of the little girl's death, another child in the same family, aged two years, was smitten, and died on the third day after the attack; the parents refusing to employ medical aid; but I am told by the father, a gentleman of intelligence, that the symptoms in this case were precisely similar to those in the case detailed above, differing only in the rapidity of their succession.

This case illustrates fully ten others that came under my treatment, only two of which recovered.

I have a most painful recollection of a like epidemic (?) of Pneumonia and Cerebro-Spinal Meningitis, that occurred in this locality in the Spring of 1863, having, at that time, lost an only child by it. At that time it was also extremely fatal, not only in my own practice, but in that of my colleagues. All of the children attacked with this disease in 1863, and also in 1870, were less than ten years of age.

A curious circumstance, which I may mention in this connection, is the fact that, following both these epidemics, Pneumonia, in an exceedingly fatal form, attacked many of the lower order of animals, especially the horse; while at the same season, in other years, the disease among animals is comparatively unknown.

